# Infrequent small bowel intestinal bacterial overgrowth in malnourished Zambian children

**DOI:** 10.11604/pamj.2020.35.29.9831

**Published:** 2020-02-07

**Authors:** Namwiinga Ndulo, Rory Peters, Kirsten Macgregor, Mercy Imasiku, Beatrice Amadi, Paul Kelly

**Affiliations:** 1 Tropical Gastroenterology & Nutrition group, University of Zambia School of Medicine, Lusaka, Zambia; 2 Blizard Institute, Barts & The London School of Medicine, Queen Mary University of London, 4 Newark Street, London E1 2AT, UK

**Keywords:** Malnutrition, dysbiosis, small intestinal bacterial overgrowth, Africa

## Abstract

There is evidence that children with malnutrition have an increased frequency of small intestinal bacterial overgrowth (SIBO) due to impaired gastric acidity, impaired intestinal motility, and dysbiosis. Children with malnutrition respond to antibiotic therapy but it is not clear if this effect is mediated by treatment of SIBO. We set out to determine the frequency of SIBO in children of varying nutritional status in a poor community in Lusaka, Zambia. Hydrogen breath testing, following a dose of 1g/kg oral glucose, was used to determine the presence of SIBO amongst the study participants. Forty nine children, 45 of whom had varying degrees of malnutrition, completed a full series of observations at 15, 30 and 60 minutes. Four children (8%) had a rise of 10ppm from baseline, consistent with SIBO. No correlation with nutritional status was observed. In this small study of Zambian children, SIBO was infrequent and unrelated to nutritional status.

## Introduction

Globally, 53% of child deaths worldwide are attributable to malnutrition [1] and HIV co-infection is associated with further increases in mortality. Malnutrition in early life is also associated with significant long-term morbidity, reduced socioeconomic status and cognitive ability. While significant advances have been made in management of severe acute malnutrition, prevention of stunting has remained relatively intractable [2]. Small intestinal bacterial overgrowth (SIBO) has been associated with malnutrition for several decades [3]. It is likely to reflect reduced intestinal clearance, gastric hypochlorhydria [4] and systemic immunodeficiency. The gold standard for diagnosis is culture of jejunal aspirates; a bacterial colony count ≥10^5^CFU/ml is characteristic of SIBO. However less than 30% of the bacteria present in the gut microflora are culturable [5], and this approach requires an invasive procedure. So, recently, studies have tended to use breath tests [6] or retrospectively diagnose SIBO following improvement with empirical treatment. An early study of Jamaican malnourished children [7] showed that the administration of antibiotics improved growth. More recently there have been attempts to characterise the overgrowth using DNA sequencing, but hydrogen breath testing is attractive for paediatric research. We aimed to test the hypothesis that there would be a high prevalence of bacterial overgrowth in malnourished children in our population and that it would be correlated with severity of malnutrition.

## Methods

This study was carried out from January-May 2014. Study participants aged 0-60 months were recruited from a nutrition screening programme in an impoverished community in Lusaka, Zambia. Written consent was obtained from the parent or guardian of study participants after a verbal explanation in a preferred local language, supported by a written information sheet. Approval was obtained from the University of Zambia Biomedical Research Ethics Committee (approval 003-02-12). Inclusion criteria were met if the participants were resident in the catchment area of the nutrition screening programme, with WHO weight for age z-score less than -1. Participants were excluded from the study if any of the following criteria were met: 1) complicated malnutrition requiring inpatient hospital care; 2) antibiotic treatment within four weeks prior to study enrolment; 3) laxative treatment within four weeks prior to study enrolment; 4) known or suspected postprandial hypoglycaemia. Measurements of exhaled breath hydrogen were made, at baseline and 15, 30 and 60 minutes following consumption of a test glucose load, using a portable Gastro Plus breath hydrogen analyser (Bedfont Scientific, Maidstone, Kent, UK). To avoid pre-test fasting a baseline breath hydrogen concentration of less than 12ppm was required for inclusion. The test glucose load was in the form of a 1g/10ml solution administered orally at a dose of 10ml/kg, then no additional feeding was permitted for one hour. A positive test was defined as a rise in breath hydrogen concentration of ≥10ppm above the participant’s baseline value. In addition to the breath test measurements, anthropometric measurements (weight for age (WAZ), height for age (HAZ) and weight for height (WHZ) z scores and mid upper arm circumference (MUAC)) were made. Participants’ HIV status, recent diarrhoea and the presence of oedema or skin lesions were recorded. Those participants with SIBO were then treated using a combination therapy of metronidazole and co-trimoxazole.

## Results

A total of 51 participants were recruited with the consent of a parent or guardian; these children displayed a wide variety of nutritional status (Table 1). Two participants were excluded from the final analysis having failed to complete all the required breath tests, so 49 participants were included, with varying degrees of malnutrition (Table 2). Within the cohort only a single patient was found to have serological evidence of HIV infection. Of the 49 participants, 4 (8.2%) had positive breath tests. Malnourished and non-malnourished children did not differ in the prevalence of SIBO (Table 2). Three of the four (75%) participants positive for SIBO had received amoxicillin at the clinic, though this pre-dated the four week antibiotic free window stipulated in the study inclusion criteria. Of the non-SIBO participants, 17 of 45 (38%) participants had received amoxicillin prior to the exclusion period (P=0.29).

**Table 1:** Characteristics of children by sex

	Boys	Girls	*P*
n	20	29	
Age (months) (median, IQR)	17 (14,23)	15 (12,20)	0.24
WAZ (<-3, <-2, <-1, <median)	11,6,2,1	15,12,2,0	0.60
HAZ (<-3, <-2, <-1, <median)	14,3,3,0	18,6,4,1	0.95
WHZ (<-4, <-3, <-2, <-1, <median)	1,2,5,7,5	2,4,11,5,7	0.71
Oedema present	14	16	0.38
Recent (<4 weeks) amoxicillin use	8	12	1.00
Recent (<4 weeks) mebendazole use	10	11	0.56

**Table 2:** Nutritional status and presence or absence of SIBO

	SIBO	No SIBO	Total	*P*
Weight for age z score				
≤ Med	0	1	1	
≤-1	1	3	4	
≤-2	1	17	18	
≤-3	2	24	26	
≤-4	0	0	0	
Total	4	45	49	0.66
Height for age z score				
≤ Med	0	1	1	
≤-1	1	6	7	
≤-2	1	8	9	
≤-3	2	30	32	
≤-4	0	0	0	
Total	4	45	49	0.48
Weight for Height z score				
≤ Med	1	11	12	
≤-1	1	11	12	
≤-2	1	15	16	
≤-3	0	6	6	
≤-4	1	2	3	
Total	4	45	49	0.58

## Discussion

This study revealed a low prevalence (8%) of SIBO in children with malnutrition in Lusaka. Although hydrogen breath testing is non-invasive, children require considerable reassurance in order to tolerate a face mask for the few minutes required for the test, and it is not easy to study large numbers of children. Although we were unable to robustly test our hypothesis that SIBO would correlate with the severity of malnutrition due to the small numbers tested, our data do not appear to support that hypothesis. In this small sample, we were unable to recruit enough children to test the hypothesis that HIV might be associated with increased frequency of SIBO, and with the low prevalence in HIV-infected children the sample size for such an analysis would be large. There is currently much interest in the changes in the microflora in human disease and in predisposition to disease, interest which is only possible thanks to new DNA sequencing technologies [5, 8]. Recent data also indicate that dysbiosis may characterise malnutrition in children with kwashiorkor in Malawi [8], possibly through effects on nutrient absorption and intestinal permeability [9]. SIBO may reflect the tip of an iceberg of changes in the microflora. While is it almost certainly an insensitive and crude way of analysing changes in the composition of the microflora, it probably reflects a shift in the metabolome-the grand total metabolic unit of host and flora-largely in the colon. More detailed studies of metabolomic disturbances over the coming years can be expected to reveal how SIBO affects host physiology. Reassuringly, SIBO remains treatable with simple antibiotics [10].

## Conclusion

In this small study, SIBO was found in 8% of Zambian children living in a poor community, and was not related to nutritional status.

### What is known about this topic

- Small intestinal bacterial overgrowth (SIBO) can complicate severe malnutrition;
- Antibiotics are known to contribute to improved recovery from malnutrition;
- It is not known if treatment of SIBO explains the beneficial effect of antibiotics.

### What this study adds

- The prevalence of SIBO in this community was low at 8%;
- No relationship was detected with nutritional status.
